# Electrical Resistivity and Tensile Strength Relationship in Heat-Treated All Aluminum Alloy Wire Conductors

**DOI:** 10.3390/ma14195738

**Published:** 2021-10-01

**Authors:** Nidal Alshwawreh, Baider Alhamarneh, Qutaiba Altwarah, Shamel Quandour, Shadi Barghout, Osama Ayasrah

**Affiliations:** 1 Department of Industrial Engineering, German Jordanian University, P.O. Box 35247, Amman 11180, Jordan; qutaiba.altouri@gju.edu.jo; 2 Department of Mechanical and Maintenance Engineering, German Jordanian University, P.O. Box 35247, Amman 11180, Jordan; bayderh@gmail.com (B.A.); shamelquandour@hotmail.com (S.Q.); barghoutshadi7@gmail.com (S.B.); osamaayasrah@hotmail.com (O.A.)

**Keywords:** heat treatment, precipitation hardening, electrical resistivity, all aluminum alloy conductors, Al–Mg–Si alloys

## Abstract

Thermal processing of all aluminum alloy conductors (AAAC) is an important step that is performed to enhance the electrical and mechanical properties after the drawing process. In these 6xxx alloys, mechanical strength and electrical conductivity are normally two mutually exclusive properties. With the increased demand for high performance power conductors, it is important to understand and control microstructural evolution processes (e.g., recovery and the formation of nanoscale precipitates) in these alloys for better electrical and mechanical characteristics. In this study, heat treatment was performed on as-drawn 6201 AAAC wire conductors. The variations in tensile strength and electrical resistivity were quantitatively studied as a function of both the treatment temperature and holding time. Two wire diameters commonly used in the manufacturing of medium and high voltage power cables were used: 1.7 mm and 3.5 mm. From the obtained data, significant changes in the electrical resistivity and tensile strength were observed with increasing the treatment time. For both wire diameters, it was observed that the correlation between strength and resistivity can be described by a simple exponential relationship. This link could be useful in predicting mechanical strength by monitoring electrical resistivity variations during industrial heat treatment of AAAC wire conductors.

## 1. Introduction

Aside from their extensive applications in the automobile industry, aluminum alloys are widely used as a medium to transport electric power due to their high electrical conductivity, superior resistance to corrosion, and their attractive strength to weight ratio. With the rising demand for electric power, the development of efficient and durable wire conductors becomes important. This can be attained by engineering the alloy’s microstructure through thermal processing in order to obtain wires with high electrical conductivity and superior mechanical strength [1,2,3,4,5]. High electrical conductivity is essential to reduce power loss during transmission while considerable mechanical strength is needed to sustain operational and seasonal mechanical loads.

All aluminum alloy conductors (AAAC) are manufactured by cold drawing to their desired final diameters. There are many studies available in literature aiming to optimize the mechanical and electrical properties of AAACs [6,7,8,9,10]. As typical reductions in the cross-section can exceed 85%, the tensile strength of these metal alloys increases due to work-hardening. In addition, the effect of drawing on electrical conductivity can be explained by the fact that metallic alloys are sensitive to the microstructural details including dislocation density, grain boundaries, and the presence of solute atoms. Thus, a dedicated heat treatment step is conventionally required after drawing to optimize both electrical and mechanical properties. In doing so, several parameters need to be considered when designing the heat treatment scenario (e.g., initial composition of the alloy and level of cold work) [7,11,12]. It is well known that microstructure evolution processes including recovery, recrystallization, grain growth, and second phase precipitation can occur during heat treatment and artificial ageing of metallic alloys. During recovery, there is a partial annihilation of dislocations resulting in some mechanical softening. In recrystallization, new dislocation-free grains form and grow on the expanse of the deformed grains leading to enhanced ductility and lower mechanical strength. Grain growth normally follows recrystallization and results in an increase in the average grain size and a reduction in grain boundaries density.

Heat treatable 6201 aluminum alloy is now widely used to produce power cables. In ternary Al–Mg–Si alloy, a sequence of precipitation events occurs where Mg and Si atoms diffuse out of the saturated solid solution to eventually form incoherent Mg_2_Si precipitates [2,13]. This process affects electrical conductivity by decreasing the contribution of solute atoms to the total electron scattering [14,15]. It also affects mechanical strength since nanoscale precipitates can significantly hinder the mobility of dislocations. In aluminum alloys, precipitation hardening can occur at room temperature (natural ageing) and can be accelerated at a relatively elevated temperature (artificial ageing). There are studies reported in literature on the effect of cold drawing on the tensile strength and electrical resistivity of aluminum alloys. For example, Quainoo and Yannacopolos [16] observed that the amount of prior cold work on AA6111 has a strong influence on the activation energy of precipitate formation resulting in an accelerated precipitation kinetics. A similar conclusion was obtained by Lan et al. [14] on 2A14 aluminum alloy. In addition, it appears that the kinetics of phase transformation during artificial ageing can be affected by prior natural ageing [17,18,19,20]. In this regard, Xin et al. [4] studied the effect of prior natural ageing on the properties of samples after artificial ageing. The research has indicated that a prior natural ageing following solution treatment results in a devastating effect on mechanical properties. In a recent study [21], it was found that the chemical composition in terms of the amount of Mg and Si plays an important role in determining the mechanical and electrical properties of 6101 wire rods. In addition, it was concluded that the best temperature and time for artificial ageing was 150 °C and 4–10 h, respectively.

Several characterization techniques are commonly used in the literature to quantify the kinetics of precipitate formation and its effect on mechanical properties. The investigation of precipitate formation and microstructure evolution is a complex process and normally requires using a combination of characterization techniques including differential scanning calorimetry (DSC) [22,23], atom probe [12,24], positron annihilation lifetime spectroscopy [17,18], electron backscatter diffraction [8,25], transmission electron microscopy [2,4,5], and electrical resistivity measurements [26,27,28,29]. While these techniques can reveal various aspects of the microstructure evolution processes and precipitate details, electrical resistivity measurement has many advantages compared to these techniques. Firstly, the electrical resistivity measurement is relatively easy to perform and does not require a sophisticated setup. Secondly, the measurement time and sample preparation are minimal, which can be an advantage from a practical viewpoint. While DSC can detect the onset and completion of the precipitation process, the sample volume is often too small. On the other hand, electrical resistivity measurements can provide a more representative information from a relatively larger sample volume, which improves the accuracy of the data [29,30]. Moreover, electrical resistivity is very sensitive to any variations in solute atoms’ concentration that may occur in the alloy as a result of the precipitation reaction. In the case of AAAC wires, electrical resistivity is not only a “sensor” for precipitation kinetics but it also represents an important parameter that needs to be minimized for high-quality power conductors [15,28].

In industrial settings where thermal processing of power conductors is often performed inside very large furnaces, accurate control of the electrical and mechanical properties is important especially when a uniform temperature distribution in the furnace is not always maintained. In this research, the effect of annealing parameters in terms of temperature and holding time on the tensile strength and the electrical resistivity of as-drawn 6201 Al–Mg–Si conductors was studied. The idea was to examine the link between the two properties after drawing and heat treatment in a simulation of actual industrial practice. From the obtained results, the correlation between electrical resistivity and tensile strength was analyzed. Furthermore, the relationship between the measured parameters was investigated on two widely used wire diameters in the manufacturing of electrical power cables (i.e., 1.7 mm and 3.5 mm).

## 2. Materials and Methods

The 1.7 mm and 3.5 mm in diameter wires were obtained by cold drawing in which a 9.5 mm in diameter rod was passed through a series of converging dies. These 9.5 rods were produced commercially with their chemical composition indicated in Table 1. The average ultimate tensile strength of these rods was 212 MPa while their electrical resistivity was 50% IACS, both measured at room temperature. For a diameter of 3.5 mm, the rods were drawn in seven stages resulting in a cross-sectional area reduction of 86.4%. On the other hand, for a diameter of 1.7 mm, the rods were drawn in 14 stages resulting in a cross-sectional area reduction of 96.8%. The as-drawn wires were cut into 20 cm samples and then tested for their mechanical and electrical properties. This was performed at room temperature (25 °C) and after heat treatment (130–250 °C for 10–360 min). The heat treatment was conducted in a closed-loop temperature-controlled oven (Heratherm OGS60, Thermo Fisher Scientific, Waltham, MA, USA,). In the heat treatment cycle, the temperature was ramped from 25 °C to the desired temperature for the specified annealing time. In these experiments and in order to ensure accuracy of the electrical and mechanical measurements, three samples from different sections of the drawn wire were heat treated at the same conditions. After thermal treatment was complete, all specimens were air-cooled outside the furnace for one hour. Then, the average tensile strength of these samples was measured at room temperature employing a universal materials testing machine (Testometric FS300CT, Rochdale, UK) equipped with 2500 kg load cell (type DBBMTCL) at a deformation rate of 80 mm/min. The average gauge length of the tested samples was 60 mm and 80 mm for the 1.7 mm and 3.5 mm in diameter wires, respectively.

**Figure 1:**
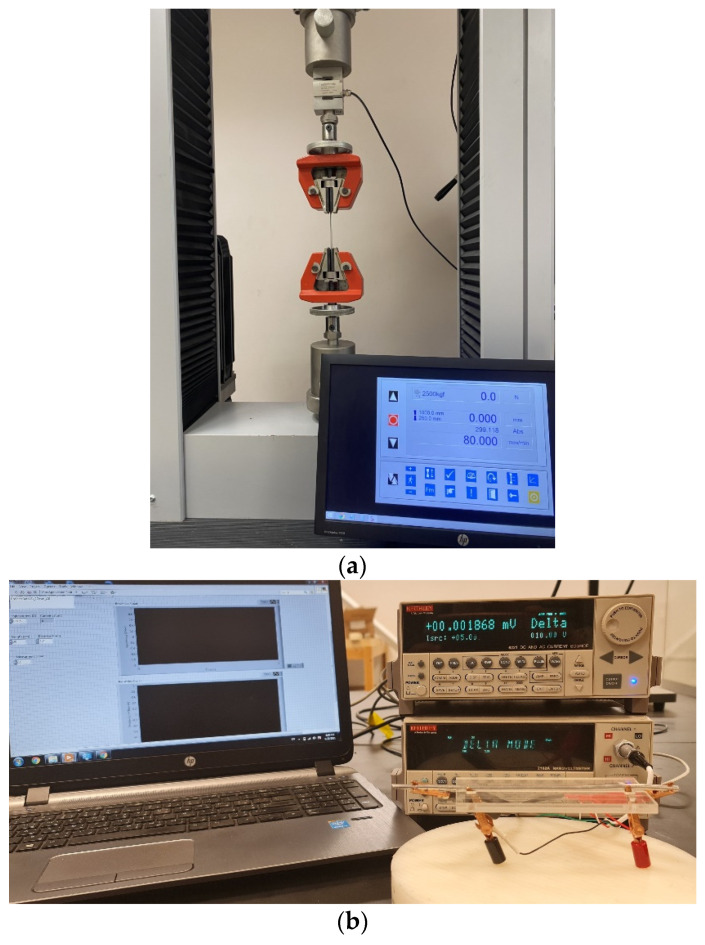
(**a**) The tensile testing setup showing the gripped 3.5 mm wire. The test was performed at room temperature via software to control the crosshead speed; (**b**) in-house electrical resistivity setup utilizing a current source and nanovoltmeter connected in Delta mode. The LabVIEW-controlled resistivity test was performed using the four-point method.

Electrical resistivity measurements were performed by the four-point method employing a Keithely 6221 DC current source and a Keithely 2182A nanovoltmeter. In order to suppress the thermoelectric noise, the two instruments were connected in Delta mode where the current polarity is alternated while the nanovoltmeter measures the potential drop at each polarity. Electrical resistivity (ρ) was then obtained from current and voltage values by accounting for the sample cross-section and length where:

(1) $$\rho =\frac{VA}{IL}$$

here, V represents the measured voltage, A is the cross-sectional area, I donates the injected current, and L is the sample length. During all resistivity measurements, the current passing through each sample was set to 5 mA. The measurements data were captured by a LabVIEW code that records the average of 10 Delta-mode measurements per sample. The tensile and resistivity testing setups are shown in Figure 1.

## 3. Results

### 3.1. Tensile Strength

**Figure 2:**
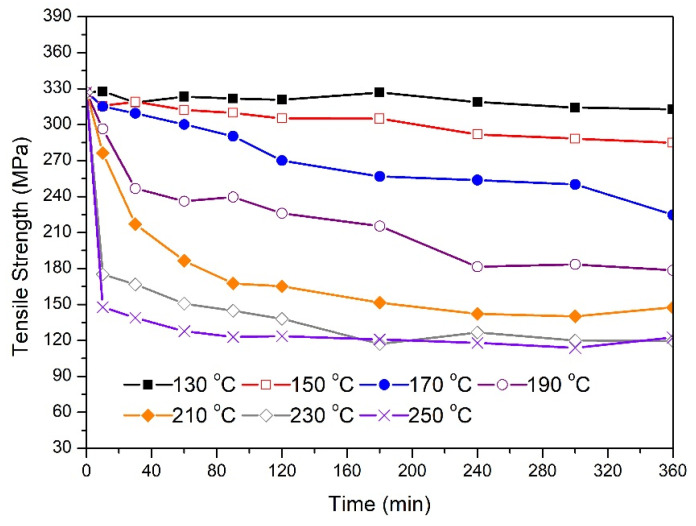
Changes in tensile strength as a function of the annealing time for 1.7 mm samples at the specified temperature.

Figure 2 shows the effect of the annealing time on the tensile strength for 1.7 mm in diameter wires treated at different temperatures. While the average tensile strength of as-drawn samples was 327 MPa, heat treatment can decrease this value dramatically. The main heat treatment parameters that have an effect on mechanical properties of metals and alloys are the treatment temperature and holding time. Here, there was no significant change in tensile strength for samples treated at 130 °C during the entire six hour treatment period. However, for temperatures above 130 °C, there was a significant drop in tensile strength values. It can be shown that as annealing time increased, the tensile strength generally decreased. With an as-drawn sample as a reference, about a 13% decrease in tensile strength was measured for samples aged at 150 °C after six hours of treatment. As the temperature increased, the percentage reduction in tensile strength at the end of the thermal treatment increased. A final tensile strength of around 120 MPa was achieved for samples treated at 230 °C and 250 °C, leading to around a 63% total drop. While a gradual decrease in tensile strength for samples aged at 150 °C, 170 °C, and 190 °C was observed, a sharp drop in strength was detected during the first 10 min of thermal treatment at 230 °C and 250 °C. Following this fast drop, samples treated at 250 °C did not experience any significant change in tensile strength after about one hour in the furnace.

**Figure 3:**
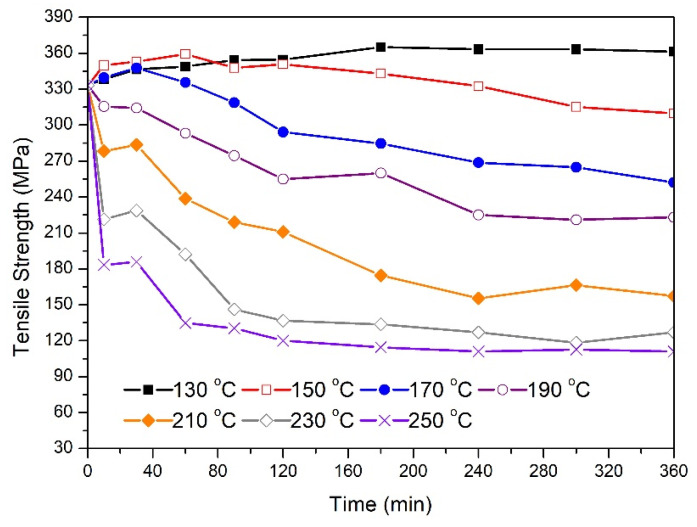
Changes in tensile strength as a function of the annealing time for 3.5 mm samples at the specified temperature.

Figure 3 shows the tensile strength data for 3.5 mm in diameter wires treated at different temperatures. The as-drawn samples had an average tensile strength approximately similar to that of 1.7 mm samples (333 MPa). From the shown data, wires treated at 130 °C experienced an increase in their tensile strength reaching 365 MPa after three hours in the furnace. No further increase was observed for the remaining treatment time. On the other hand, and similar to the behavior depicted in Figure 2, there were remarkable variations in the strength-time profiles as the temperature exceeded 130 °C. Here, the percentage reduction in tensile strength at the end of the treatment increased with ageing temperature (150–250 °C). It is observed that samples treated at 150 °C and 170 °C reached a tensile strength peak in the early stage of thermal processing. This peak was not detected in other samples treated at higher temperatures (190–250 °C). Similar to the behavior of the 1.7 mm wires, a rapid drop in the tensile strength for samples treated at 230 °C and 250 °C was detected during the first 10 min of heat treatment. Compared with the corresponding data presented in Figure 2, there are some differences in the strength-time profiles during the first hour of treatment at temperatures above 190 °C. While the 1.7 mm wire experienced a sharp and continuous drop at these temperatures, the 3.5 mm samples experienced slight or no change in strength during 10 to 30 min of annealing. After that, the tensile strength resumed its drop with time. The relative change in tensile strength for 3.5 mm and 1.7 mm samples after six hours of treatment at 250 °C was approximately identical (67% and 63%, respectively).

### 3.2. Electrical Resistivity

**Figure 4:**
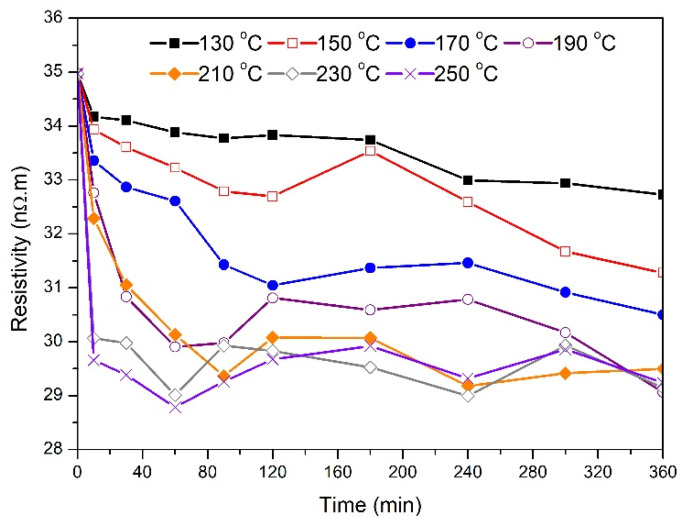
Changes in electrical resistivity as a function of the annealing time for 1.7 mm samples at the specified temperature.

Figure 4 shows the effect of the annealing temperature and holding time on the electrical resistivity of the 1.7 mm wires. The average resistivity of the as-drawn samples was around 35 nΩ.m. Samples treated at 130 °C experienced about a 7% reduction in their electrical resistivity at the end of the treatment compared to the as-drawn samples. Similar to the behavior observed in the tensile strength data reported above, the electrical resistivity of wires annealed at 230 °C and 250 °C dropped drastically during the first 10 min. The lowest final electrical resistivity value (≈29.5 nΩ.m) was attained by samples treated at 190 °C, 230 °C, and 250 °C. For samples annealed at 150 °C, and after a gradual drop in resistivity to around 32.7 nΩ.m, the resistivity increased to 33.5 nΩ.m before resuming its drop to a final value of 31.3 nΩ.m. A similar resistivity increase in the middle of the treatment was also observed for samples treated at 170 °C and higher. However, there was a relative final drop in resistivity at the end of the treatment that ranged from 7% to 16%.

**Figure 5:**
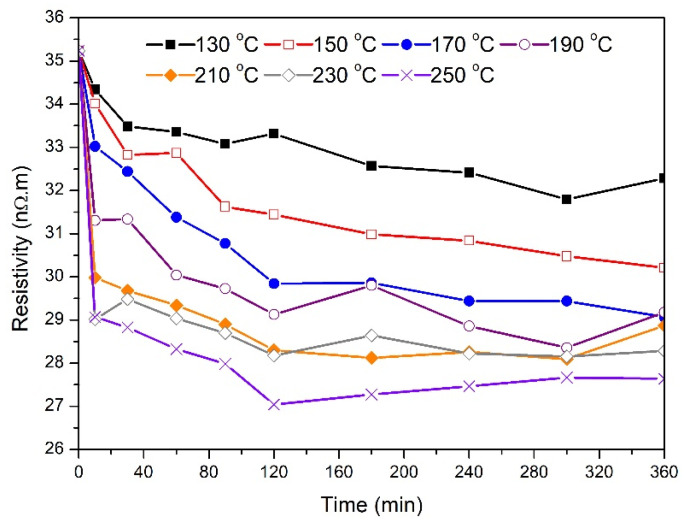
Changes in electrical resistivity as a function of the annealing time for 3.5 mm samples at the specified temperature.

The effect of the annealing temperature and holding time on the electrical resistivity of the 3.5 mm wires is illustrated in Figure 5. The average electrical resistivity of the as-drawn samples was not very different from that of the 1.7 mm samples. For samples treated at 130 °C, 150 °C, and 190 °C, there was a gradual decrease in electrical resistivity with annealing time. As the annealing temperature increased, the percentage drop in the electrical resistivity at the end of the treatment generally increased. While a final resistivity of about 29 nΩ.m was achieved by samples annealed at 170 °C, 190 °C, and 210 °C, the largest reduction in electrical resistivity was obtained for samples annealed at 250 °C (about 22% compared to untreated samples). Similar to the 1.7 mm electrical resistivity data presented in Figure 4, the data also indicated that wires annealed at 210 °C, 230 °C, and 250 °C experienced a rapid drop in electrical resistivity values during the first 10 min of thermal treatment.

## 4. Discussion

The observed changes in tensile strength and electrical resistivity of 6201 AAAC wires were influenced by a combination of two processes: precipitation hardening and annealing. In precipitation hardening of Al–Mg–Si alloys, regions in the supersaturated solid solution α are converted to solute atom clusters (spherical in shape and known as G.P. zones) and then to fine needle-shaped metastable coherent precipitates β″. The full transformation reaction is well documented in the literature [23,31] and can be represented by:

α → G.P. zones → β″ → β′ → β

here, β″ precipitates transform to rod-shaped β′ and eventually to the equilibrium phase of incoherent β (Mg_2_Si). Some reports in the literature indicated the possibility of forming an equilibrium Si phase at the end of the sequence, which appeared to be controlled by the Mg to Si ratio [7,11,12]. The reaction sequence also depends on the composition of the alloy and can be influenced by the presence of Cu in comparable quantity to that of Mg and Si. In this case, Cu-containing precipitates form such as lath-shaped Q^′^, which appears after β″ and is followed by the formation of equilibrium Q + Mg_2_Si phases [31]. It is well-known that the strengthening starts as soon as the G.P. zones are formed in as-quenched solution-treated specimens. However, β″ nanoprecipitates are responsible for attaining maximum mechanical strength (peak-aged conditions). Since heat treatment was performed on as-drawn samples in this study without solution treatment, no strengthening was observed in most of the samples during the entire treatment time. This means that the observed reduction in tensile strength with ageing was caused by a possible combination of static recovery processes, precipitation of β′, and coarsening of β″, in addition to any possible formation of an equilibrium phase. During recovery, some of the internal stored energy from cold work is released after partial dislocation annihilation. Since recovery is a thermally activated process driven by dislocations density, temperature has a significant effect on the microstructure and the resulting mechanical properties. Moreover, the ageing temperature affects the diffusion of solute atoms, which occurs during the formation of various precipitates. It is proposed that the dislocations originated from cold work can enhance the depletion of solute atoms through a mechanism known as pipe diffusion [24]. This mechanism influences the contribution of solute atoms to the overall alloy strength. In terms of electrical properties, and based on Matthiessen’s rule, the total electrical resistivity can be described by combined effects of lattice vibrations and imperfections on electron scattering. In this way, the electrical resistivity (ρ) can be written as:

(2) $$\rho =\rho_{T}+\rho_{gb}+\rho_{d}+\rho_{i}+\rho_{p}$$

where $\rho_{T}$, $\rho_{gb}$, $\rho_{d}$, $\;\rho_{i}$, and $\rho_{p}$ represent the contribution of temperature, grain boundaries, dislocations and vacancies, solute atoms, and precipitates to the overall electrical resistivity in polycrystalline bulk materials, respectively. Since precipitation hardening involves diffusion of solute atoms while recovery leads to a reduction in dislocation density, electrical resistivity measurement can provide valuable information regarding the kinetics of these microstructural evolution processes in heat treated AAAC wires. It is well known that the mean free path (MFP) of electrons at room temperature in aluminum is less than 20 nm [32]. This means that when the distance between the precipitates becomes larger than the MFP, their effect on electrical conductivity becomes insignificant. Moreover, the grains in aluminum alloys experience thermal stability at typical ageing temperatures, which reduces the effect of grain boundaries on electron scattering. Thus, any changes in electrical conductivity values are mainly related to the variations in the concentration of solute atoms available in the matrix. As explained in the literature [24], dislocations appear to have a second order contribution to electrical conductivity in Al–Mg–Si alloys.

The 3.5 mm samples treated at 130 °C, 150 °C, and 170 °C reached a tensile strength peak at 180 min, 60 min, and 30 min, respectively. This indicates that the raw material before drawing was not at peak-aged conditions and there existed an excess quantity of solute atoms in the aluminum matrix. The increase in strength is then attributed to the nucleation and growth of β″ precipitates in addition to those that might have formed during natural ageing and processing prior to drawing. Apparently, precipitation hardening appeared to have a greater effect on strength here than any possible softening arising from the recovery process. The fact that the 130 °C-treated wires experienced no change in tensile strength after reaching 360 MPa could be explained by a possible balance between nucleation and growth of both β″ and lath-shaped semicoherent β’ precipitates. For samples treated at 150 °C and 170 °C, the drop in strength can be attributed to a combined effect of recovery, coarsening of β″ precipitates, and the formation of β’. Here, the drop in tensile strength is related to the change in the nature of interaction between dislocations and precipitates. The effect of recovery and precipitation sequence is more visible in samples annealed at 190 °C to 250 °C as indicated by the sharp drop in electrical resistivity during the first 10 min of annealing. Here, the magnitude of this drop increased with temperature leading to a tensile strength of 180 MPa for wires annealed at 250 °C. There appeared to be a plateau in tensile strength between 10 and 30 min of annealing before dropping again. This could be an indication of β″ precipitation at these temperatures, which appears to balance the effect of recovery and the formation of β′. After 30 min of annealing, dislocation cancellation through recovery appeared to progress in parallel with an increase in the volume fraction of β′ precipitates. These microstructural changes (reduction in solid solution strengthening, precipitate formation and transformation, and annihilation of dislocations after annealing) can explain the significant drop in tensile strength after six hours of treatment, reaching values lower than 120 MPa for samples treated at the maximum temperature employed in this study (250 °C). A similar drop in mechanical strength was also observed for the aged 1.7 mm wires. Although the as-drawn 1.7 mm and 3.5 mm wires had the same tensile strength values, it appeared that the rate of tensile strength drop was affected by the percentage cold work. For example, the drop in tensile strength after one hour of ageing at 210 °C was about 45% and 31% for 1.7 mm and 3.5 mm samples, respectively. This can be attributed to the higher dislocation density available in the 1.7 mm microstructure, which appears to accelerate the pipe diffusion of solute atoms, as explained previously. This may also explain the absence of the plateau regions in the strength-time profiles of the aged 1.7 mm wires. Here, and since the activation energy for precipitation is less when high dislocation density is present, the formation of β′ is accelerated along with faster coarsening of β″ precipitates and higher recovery rate.

The changes in tensile strength were associated with variations in electrical resistivity as indicated in Figure 4 and Figure 5. For 3.5 mm wires, the initial drop in resistivity for samples annealed at 130 °C to 170 °C indicates that solute atoms are diffusing out from aluminum matrix to form β″ precipitates. While the tensile strength of the 130 °C sample remained constant for annealing time above three hours, resistivity continued to decrease until five hours, which supports the possibility that β″ and β’ precipitates are forming simultaneously. However, samples annealed at 190 °C experienced continuous decrease in resistivity during the entire six hours. In this case, most of the drop occurred during the first two hours of treatment. In all treated samples, the drop in resistivity appeared to be sharp during the first 30 min of thermal processing where the scale of this drop increased as the annealing temperature increased from 130 °C to 230 °C. A similar drop in resistivity was observed for the 1.7 mm wires indicating similar precipitation mechanism. The slight variations in the resistivity trend after about one hour of treatment is not clear, but it could be related to local redistribution of the solute atoms during thermal processing. Usually, the required minimum tensile strength for wire conductors is about 195 MPa while the maximum electrical resistivity is about 30.5 nΩ.m. For 1.7 mm, the best result was achieved at a treatment temperature and time of 190 °C and three hours, respectively. On the other hand, there are more options to obtain the desired characteristics for the 3.5 mm wires. For example, a heat treatment time for about 300 min at 150 °C was suitable while only 10 min at 210 °C appeared to be sufficient.

**Figure 6:**
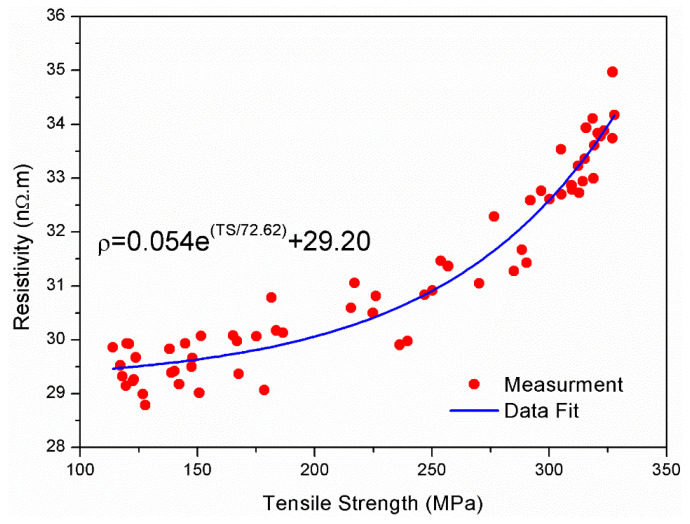
Relationship between electrical resistivity and tensile strength of 1.7 mm wires.

**Figure 7:**
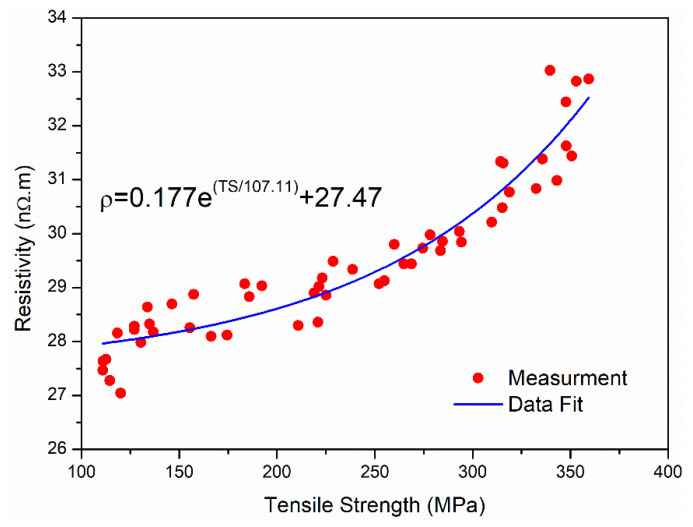
Relationship between electrical resistivity and tensile strength of 3.5 mm wires.

Figure 6 shows the relationship between electrical resistivity and tensile strength for 1.7 mm wires. The 64 points on the graph were obtained by combining the tensile strength data in Figure 2 with the resistivity data presented in Figure 4 (a total of 64 points are included where each point represents the average measurement of three samples). As tensile strength increased from about 140 MPa to about 350 MPa, the electrical resistivity increased from about 29.5 nΩ.m to about 34 nΩ.m. It appears that the relationship between resistivity and tensile strength can be expressed by a simple exponential function. From the fit of the measured data, the electrical resistivity (in nΩ.m) can be expressed mathematically as:

(3) $$\rho =ae^{\frac{TS}{b}}+c$$

where TS is the tensile strength in MPa and a, b, and c are constants. For this case, a = 0.054, b = 72.62, and c = 29.20. Figure 7 shows the same analysis for the 3.5 mm in diameter wires. It appears that the relationship between the strength and resistivity also follows the form described by Equation (3). However, the constants in the equation were different than those obtained from fitting the 1.7 mm data (a=0.177, b=107.11, and c=27.47). The obtained relationship can be useful in predicting the electrical resistivity values from tensile strength data or vice versa. This can reduce the number of samples required for routine quality check during production. The findings in this research also indicate that this relationship is valid for two most common diameters (1.7 mm and 3.5 mm). Thus, the relationship can help production engineers in evaluating the effect of annealing and ageing in AAAC alloys with a reduced number of tests. It is possible that some conditions such as alloy chemistry and extended periods of natural ageing can affect the fitting constants in the obtained model. A detailed study should be performed to quantify the effect of these parameters and validate the obtained relationship between electrical resistivity and tensile strength of the heat treated aluminum alloy samples.

## 5. Conclusions

The effect of the annealing temperature and holding time on the tensile strength and electrical resistivity of 6201 aluminum alloy wire conductors was analyzed. The study focused on 1.7 mm and 3.5 mm wires that were cold drawn and annealed without performing solution treatment after the drawing process. After testing more than 380 samples, it was observed that both the tensile strength and electrical resistivity are sensitive to the variations in the ageing process parameters. Generally, and at temperatures higher than 150 °C, it appears that as the ageing time increases, both the tensile strength and electrical resistivity decrease. It was found that the best treatment scenario for the 1.7 mm wires was heating at 190 °C for three hours. On the other hand, the 3.5 mm wires can be successfully heat treated at different temperatures ranging from 150 °C to 230 °C. From the obtained data, the nature of the correlation between the electrical resistivity and tensile strength of the wires after annealing was determined. It was proven that the two properties can be linked using a simple exponential relationship. Thus, the electrical resistivity can be reasonably predicted from the tensile strength values regardless of the heat treatment scenario. Moreover, the same trend was observed for both the 1.7 mm and the 3.5 mm wires. This study can be useful to the manufacturers of electrical wires in predicting the tensile strength from electrical resistivity, which is a nondestructive test and relatively easy to perform. Further work is required to investigate the validity of the obtained model for other wire diameters (e.g., 4.5 mm) and various alloy chemistries.

## Figures and Tables

**Table 1:** The chemical composition of the investigated aluminum alloy wires (in wt. %).

Al	Mg	Si	Fe	Cu	B
98.48	0.670	0.578	0.22	0.014	0.009

## Data Availability

The data presented in this study are available on request from the corresponding author.
